# Is the A-Chain the Engine That Drives the Diversity of C1q Functions? Revisiting Its Unique Structure

**DOI:** 10.3389/fimmu.2018.00162

**Published:** 2018-02-05

**Authors:** Berhane Ghebrehiwet, Evelyn Kandov, Uday Kishore, Ellinor I. B. Peerschke

**Affiliations:** ^1^Departments of Medicine, Stony Brook University, Stony Brook, NY, United States; ^2^Biosciences, College of Health and Life Sciences, Brunel University London, Uxbridge, United Kingdom; ^3^Department of Laboratory Medicine, Memorial Sloan-Kettering Cancer Center, New York, NY, United States

**Keywords:** Complement, classical pathway, C1q, A chain, charge pattern recognition, C1q receptor

## Abstract

The immunopathological functions associated with human C1q are still growing in terms of novelty, diversity, and pathologic relevance. It is, therefore, not surprising that C1q is being recognized as an important molecular bridge between innate and adaptive immunity. The secret of this functional diversity, in turn, resides in the elegant but complex structure of the C1q molecule, which is assembled from three distinct gene products: A, B, and C, each of which has evolved from a separate and unique ancestral gene template. The C1q molecule is made up of 6A, 6B, and 6C polypeptide chains, which are held together through strong covalent and non-covalent bonds to form the 18-chain, bouquet-of-flower-like protein that we know today. The assembled C1q protein displays at least two distinct structural and functional regions: the collagen-like region (cC1q) and the globular head region (gC1q), each being capable of driving a diverse range of ligand- or receptor-mediated biological functions. What is most intriguing, however, is the observation that most of the functions appear to be predominantly driven by the A-chain of the molecule, which begs the question: what are the evolutionary modifications or rearrangements that singularly shaped the primordial A-chain gene to become a pluripotent and versatile component of the intact C1q molecule? Here, we revisit and discuss some of the known unique structural and functional features of the A-chain, which may have contributed to its versatility.

## The Complex Structure of C1q

C1q is the first subcomponent of the complement classical pathway. In addition to its complement activation mediated immune functions, it has a broad range of developmental homeostatic functions that are not dependent on its ability to activate the classical pathway [reviewed in Ref. (1)]. The functional versatility of C1q depends on several unique structural and functional properties (1–3). It is made up of three chains, A, B, and C, which are the product of three distinct genes, found highly clustered and aligned 5′⇑3′, in the same orientation, in the order A–C–B on a 24 kb stretch of DNA on chromosome 1p at position 36.12 (4, 5). Each chain contains an N-terminal collagen-like region and a C-terminal globular head region. There are 18 chains in the intact C1q molecule: 6A, 6B, and 6C, which are arranged first as a single heterotrimeric strand comprising of A, B, and C, in which the A chain and B chain within the strand are covalently linked to each other, whereas the C-chain of one strand which is non-covalently associated with the AB dimer, nonetheless forms a covalent link with the C chain of a neighboring ABC strand to form an ABC-CBA doublet. Three such doublets are then held together with non-covalent bonds to give rise to the well-recognized hexameric structure of C1q. The globular “heads” of each ABC strand are linked *via* six collagen-like “stalks” to a fibril-like central region resulting in two unique structural and functional domains: the collagen-like region (cC1q) and the globular “heads” or domains (gC1q) (6, 7). Each of the gC1q domains is a heterotrimeric structure made up of each of the individual chains (ghA, ghB, and ghC). What has become apparently clear is the fact that each of the gh domains is capable of recognizing a gh-specific ligand independent of the other gh domains (3, 8, 9). Therefore, assuming that each of the gh domains recognizes a single target or ligand, the C1q molecule can recognize and bind simultaneously six individual molecular patterns, making it one of the most efficient, and versatile pattern recognition molecules.

The crystal structure of the heterotrimeric gC1q domain revealed a compact jellyroll β-sandwich fold similar to that of the multifunctional tumor necrosis factor (TNF) family of proteins (10, 11). This suggested that C1q not only diverged from a primordial ancestral gene template of the innate immune system that gave birth to the TNF-α and other C1q-like proteins, but also retained some of its ancestral “cytokine-like” functions (2, 10). Therefore, C1q could be considered as a prototype “complekine,” i.e., complement protein with cytokine-like activity, which is capable of mimicking some, if not all, functions of the TNF family of proteins, including the induction of cytokines (IL-6 and IL-8) and chemokines (e.g., MCP-1) that orchestrate a myriad of a rapidly expanding list of pathophysiological processes (12, 13).

There is also an abundance of clinical evidence, which shows that genetic deficiency in C1q is associated with a wide range of clinical syndromes closely related to SLE, with rashes, glomerulonephritis, and CNS disease as well as other autoimmune diseases (14). In addition, C1q also can have a major role in tumor growth and progression (15–19). The role of C1q, being a part of tumor microenvironment, has appeared to be complex so far. In some reports, it has been shown to be protumorigenic (15–17), whereas there are recent reports of antitumor activities of C1q in the case of prostrate (18) and ovarian cancers (19).

Although individuals with congenital C1q deficiency constitute only a small cohort of patients, this strong association nonetheless implicates an important role for complement in general, and C1q in particular, in the development of SLE and other autoimmune diseases (20–24). What is perplexing, however, is the fact that among the C1q deficiencies, the A-chain of C1q should take center stage in significance as homozygous deficiency or mutation in the A-chain is almost invariably associated with various diseases (20–24). The mutation in the A-chain is due to a homogeneous mutation in which the C to T transition in codon 186 of exon 2 results in Gln-to Stop (Q186X) substitution. The question is: what are the structural signatures that make the C1q-A chain such a powerful susceptibility biomarker of these diseases? It is worth noting that although the most prevalent mutation is the C1qA, Gln208X mutation, there are other mutations in B and C chains too (25).

## Structural and Functional Characteristics of the C1q A-Chain

The genes encoding the three chains of C1q are highly conserved from zebrafish to human. Phylogenetic analysis also intimates that the C1qA, C1qB, and C1qC may have originally been generated by gene duplications from a single copy of an ancestral C1qB gene, since the latter is found in the same branch as amphioxus C1q, which is an earlier lower vertebrate than zebrafish (26). Furthermore, the IgG binding properties between fish and mammalian C1q show no difference since substitution of human C1q by fish C1q has the same activity, suggesting that the IgG or IgM recognizing properties have remained conserved throughout the evolutionary history (26, 27). However, more recent studies have shown that there is a preferential binding of the gC1q modules when it comes to IgG binding. Whereas the gC1qA (or ghA) module binds aggregated IgG and IgM in a similar manner, gC1qB (ghB) binds aggregated IgG in preference to IgM (28). The functional preferences of the gC1q domains, therefore, may reflect an evolutionary structural adaptation that resulted in recent history.

**Figure 1:**
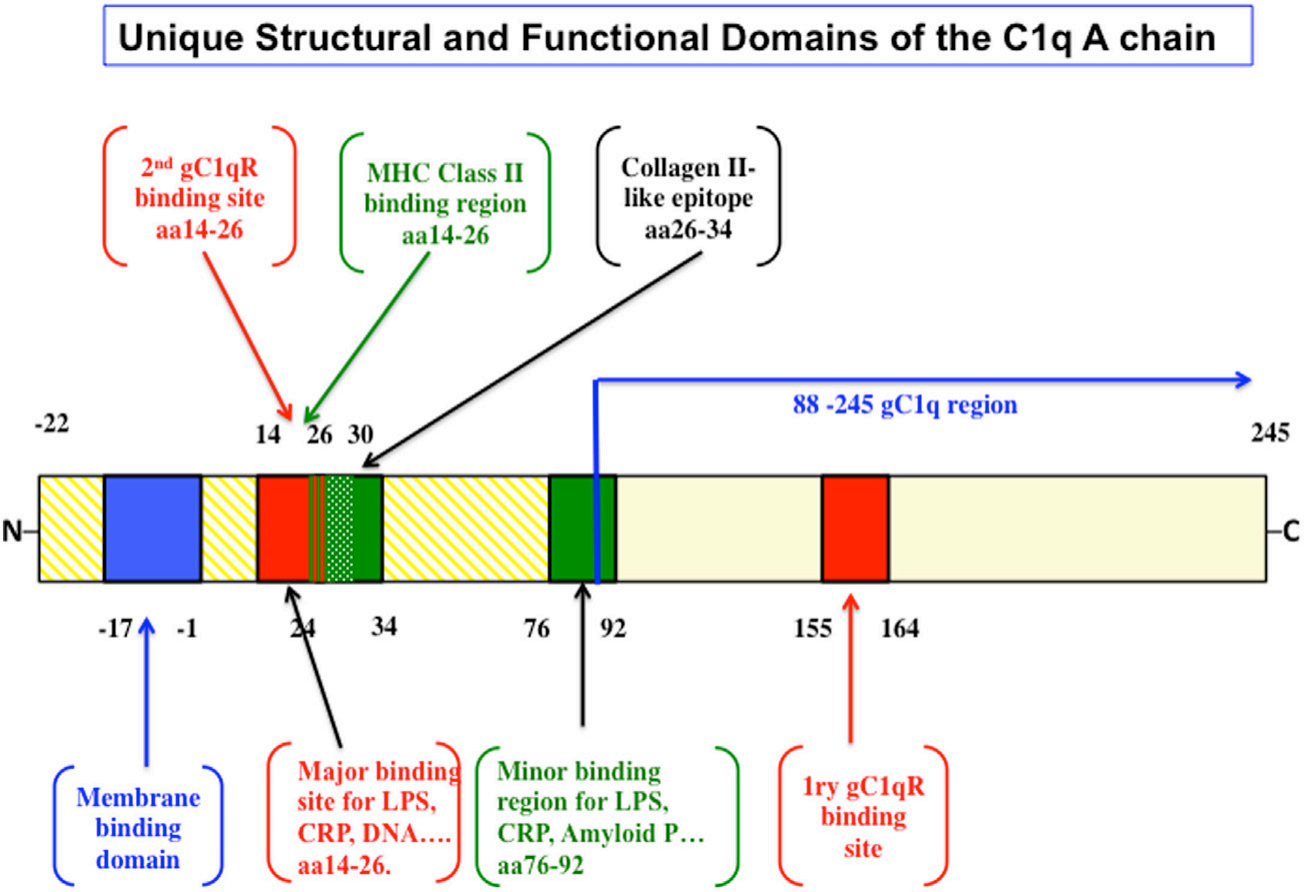
The structural and functional correlates of the C1q A-chain. The intact C1q molecule is anchored to the cell membrane by a leader peptide in the A chain. The major and minor ligand bind sites as well as the putative MHC class II binding domain are highlighted. Although the major gC1qR-binding domain spans residues 155–164, unexpected sites in the promiscuous collagen domain spanning residues 14–26 and another at 76–92 have also been shown very recently [the figure is adapted from Ref. (29)].

In an elegant and in depth review, Trinder et al. (29) analyzed the structural and functional correlates that distinguish the A-chain from the B- and C-chains. *First*, while the B and C chains are highly conserved, the A-chain is not. This fact alone should support the notion that the A-chain developed to be functionally adaptable throughout evolution. *Second*, various types of cells including macrophages and dendritic cells among a long list of others, synthesize the C1q molecule. The cell-associated molecule in turn, is anchored in the membrane *via* a 22 amino acid long leader peptide, which is found only in the A-chain (29). *Third*, the A-chain contains several antigen recognition sites (Figure 1), but in particular, possesses one major (aa 14–26) and one minor (aa 76–92) promiscuous region (29), which serve as a binding site for a wide range of non-immunoglobulin antigens including lipopolysaccharide, C-reactive protein (CRP), DNA, heparin, fibronectin, monosodium, urate crystals, amyloid P component, von Willebrand factor (30) as well as bacterial and mitochondrial membranes (29–42). Importantly, this A chain region has also been shown to bind specifically by SLE patients’ sera compared to serum derived from healthy control (43). Although recent studies have suggested that the interaction site for CRP is located in the gC1q rather than the cC1q (44–46), it is plausible to assume that certain molecules could actually bind to multiple regions of the A-chain. Regardless, these non-immunoglobulin antigens have been shown to activate the classical pathway by binding to the cC1q region of the A-chain rather than to the globular heads (29–42). In addition, the A-chain contains a collagen-type II-like sequence comprising of residues 26–34, which has been shown to suppress collagen type-II-induced arthritis in a mouse model (47). Interestingly, this same region is also predicted to be a potential MHC class II binding site (48). However, little is known about the significance of this finding but may have potential implications in autoimmunity and tolerance (48), especially since C1q has been shown to keep monocytes in a predendritic or immature phenotype, thus ensuring that unwarranted DC-driven immune response does not occur, a fact that is relevant to the development of SLE (49). Finally, although it is found only in the mouse, and not in the human C1q A-chain, the presence of an RGD sequence may also explain why human C1q still retains its ability to support adhesion of normal endothelial cells and fibroblasts (50–53) in a manner that is inhibited by an RGD peptide but not an RGE (51). Very recently, Agostinis et al. have shown that C1q can act as a bridge between hyaluronic acid (HA), an abundant matrix component of the tumor microenvironment, and the HA receptor on tumor cells, i.e., CD44, thus inducing considerable proliferation of primary tumor cells derived from malignant pleural mesothelioma (MPM) (17). Curiously, the A-chain of the globular region of C1q bound specifically and differentially to a range of LPS-free HA, leaving C-chain to liaise with MPM cells.

Thus, although it may be overly simplistic to suggest that the A-chain is the functional anchor of the C1q molecule, it appears to be clear that of the three chains, the A-chain has singularly undergone systematic and adaptable molecular evolution. Whether the selection of the A-chain to evolve as a master orchestrator of C1q functions was by design or serendipity, or whether the B- and C-chains are also undergoing similar evolution albeit at a much slower rate, are questions still for the future.

## Author Contributions

BG and EK wrote the first draft; UK and EP revised and edited the manuscript.

## Conflict of Interest Statement

The authors declare that the research was conducted in the absence of any commercial or financial relationships that could be construed as a potential conflict of interest.

## Abbreviations

ghA, ghB, and ghC, globular heads of the A, B, and C chains of C1q, respectively; gC1q, the globular heads of C1q; cC1q, the collagen domain of C1q; gC1qR, receptor for gC1q; cC1qR, receptor for cC1q; CRT, calreticulin (another name for cC1qR).
